# Safety of YAG laser vitreolysis for intraocular tissues: analysis of postoperative complications

**DOI:** 10.1007/s10792-023-02858-0

**Published:** 2023-09-04

**Authors:** Jiannan Liu, Hao Wang, Wei Gu, Tingting Zhao, Wenxue Fan, Shaomin Peng

**Affiliations:** 1 Aier School of Ophthalmology, Central South University, No. 188 Furongnan Road, Changsha, Hunan China; 2 Department of Ophthalmology, Harbin Aier Eye Hospital, Harbin, Heilongjiang, China; 3 Aier Retina Institute, Changsha, Hunan China; 4 Department of Ophthalmology, Beijing Aier-Intech Eye Hospital, Beijing, China; 5 Department of Ophthalmology, Hebei Eye Hospital, Xingtai, Hebei China; 6 Department of Ophthalmology, Dongguan Aier Eye Hospital Affiliated to Jinan University, Dongguan, Guangdong China

**Keywords:** YAG laser vitreolysis, Vitreous floaters, Floaters ablation, Vitreous opacities

## Abstract

**Purpose:**

To evaluate the safety of yttrium–aluminum-garnet (YAG) laser vitreolysis for intraocular tissues.

**Methods:**

Thirty-six New Zealand rabbits were divided as follows: Group 1000 (n = 12) treated with YAG laser of 1000 mJ (5 mJ × 200 shots), Group 2000 (n = 12) treated with YAG laser of 2000 mJ (5 mJ × 400 shots), Group 3000 (n = 12) treated with YAG laser of 3000 mJ (5 mJ × 600 shots). Either a single eye was chosen as the study eye in study groups while the other was untreated as the control group. Intraocular pressure (IOP), slit-lamp, optical coherence tomography (OCT), transmission electron microscopy (TEM), and inflammatory cytokines of aqueous humor (interleukin-1α (IL-1α), interleukin-1β (IL-1β), interleukin-8 (IL-8), and tumor necrosis factor-α (TNF-α)) were performed to examine the rabbits.

**Results:**

There were no abnormalities in the study groups of IOP, slit-lamp, and OCT examinations. Group 3000 of TEM showed: neutrophils and mitochondrial swelling on day 1, and fibroblasts and neocollagen on day 14. No abnormalities were observed in Group 1000 and 2000 of TEM. Levels of IL-1α and TNF-α increased at 12 h and decreased to baseline on day 3. Levels of IL-1β increased at 12 h and decreased to baseline on day 7. Levels of IL-8 increased on day 1 and decreased to baseline on day 3.

**Conclusion:**

YAG laser vitreolysis is safe when the distance is more than 2 mm from ablation point to the lens and the retina, and the total energy is less than 2000 mJ for one treatment procedure.

## Introduction

Vitreous is a kind of extracellular matrix, and the main component is water (99%), currently existing in a gel state^1^. The prime molecular components are fibrillar collagens and glycosaminoglycan (GAG) hyaluronan 2. Vitreous floaters are microscopic collagen fibers in the vitreous body that cast shadows on the retina, seen as floaters by patients 3. Symptomatic floaters will continue to interfere with their quality of life for some individuals 4. Floaters can be effectively treated with pars plana vitrectomy (PPV), although there are serious complications, including cataracts, macular edema, vitreous hemorrhage, and retinal detachment 5–9. In the 2016 American Society of Retinal Specialists (ASRS) Preference and Trends (PAT) survey, 25% of surgeons indicated they never conducted PPV for vitreous opacities, and 50% conducted five or fewer of these cases annually 10. Recently a growing number of surgeons are using Ultra Q Reflex™ neodymium-doped yttrium–aluminum-garnet (YAG) laser vitreolysis, on account of less invasiveness, effectiveness and higher safety for floaters treatment^11^, ^12^. It is considerably simpler to focus on the front surface of the floater with the Ultra Q Reflex™ than it is with typical YAG lasers because it has been designed to converge the operator's vision, the target illumination, and the treatment beam along the same optical path and same optical plane. Previously clinical studies have proved the efficacy and safety of YAG laser vitreolysis for symptomatic vitreous floaters, and no adverse effects were reported^12^–^14^. Yet open-angle glaucoma (OAG), cataracts, retinal tear, retinal detachment, and retinal hemorrhages occurred as unexplained complications that were described in some case reports^15^–^20^, and the majority of the reasons of the complications are unknown. In order to explore the safe energy level, we designed the study to observe the impact on intraocular tissues under different YAG laser energy levels and evaluate the safety of YAG laser vitreolysis.

## Materials and methods

A total of thirty-six adult New Zealand white rabbits (Laboratory Animal Research Center, Harbin Aier Eye Hospital) housed under standard conditions, weighing 2.5–3.0 kg with either gender, were used in this study. The rabbits were randomized into three groups (n = 12/group). Each study group received the YAG laser of different total energy: 1000 mJ (5 mJ × 200 shots) for Group 1000, 2000 mJ (5 mJ × 400 shots) for Group 2000, and 3000 mJ (5 mJ × 600 shots) for Group 3000. Either a single eye (the right eye) was chosen as the study eye in study groups while the other was untreated as the control group. All animal procedures were approved by the ethics committee of Harbin Aier Eye Hospital and carried out in strict accordance with the recommendations in the Guide for the Care and Use of Laboratory Animals of the National Institutes of Health (Approval Number: HEBAIER2019IRB09).

### Laser treatment

Rabbits were intravenously anesthetized with 3% pentobarbital sodium (1 mL/kg) and received 0.4% oxybuprocaine hydrochloride eye drops for local anesthesia. The pupil of the study eye was dilated with 0.5% phenylephrine and 0.5% tropicamide. After an Ocular Peyman 18 mm Vitreous Lens with goniosol (Alcon, USA) as a lubricating gel was applied, the vitreous ablation was performed on the study eye by using YAG laser (Ultra Q Reflex; Ellex Medical, Adelaide, Australia) in the middle of the vitreous chamber at 5 mJ per pulse.

### Clinical observation

All the rabbits were investigated by slit-lamp examination before every laser treatment and examination preoperatively and postoperatively. Intraocular pressure (IOP) measurement and optical coherence tomography (OCT) examination were performed on six rabbits randomly selected from each study group. IOP was measured preoperatively (baseline) and 30 min, 12 h, 1 day, 3 days, 7 days, 14 days, and 1 month postoperatively by the Tono-Pen AVIA Applanation Tonometer (Reichert Technologies, NY). Three recordings per eye were averaged. OCT (RIVUE-XR OPTOVUE; Optovue Inc., Fremont, CA, USA) was conducted preoperatively and 1 day, 4 days postoperatively.

### Transmission electron microscopy

Three rabbits from each study group were intravenously anesthetized with 3% pentobarbital sodium (1 mL/kg), then sacrificed by intravenous injection of air at each time point, i.e., 1 and 14 days after laser treatment, and so were the three rabbits from the control group on any day. After enucleation, the globes were opened circumferentially 3 mm behind the limbus promptly and placed in fixative solution comprising 4% paraformaldehyde for 3 days at 4 °C. Then anterior chamber angle was obtained and processed by the electron microscopy service center at Harbin Medical University following a conventional protocol. The samples were observed via transmission electron microscopy (TEM) (HT-7700, HITACHI, Japan).

### Inflammatory cytokines of aqueous humor quantification

The rest of the rabbits were intravenous anesthetized with 3% pentobarbital sodium (1 mL/kg) and received 0.4% oxybuprocaine hydrochloride eye drops for local anesthesia preoperatively (baseline) and 12 h, 1 day, 3 days, 7 days, 14 days postoperatively. 0.1 mL aqueous humor was collected from the anterior chamber with a 26 gauge needle and 1.0 mL syringe. All the samples were immediately stored at − 20 ℃ until further examination. The inflammatory cytokines of interleukin-1α (IL-1α), interleukin-1β (IL-1β), interleukin-8 (IL-8), and tumor necrosis factor-α (TNF-α) were measured using Quantibody Rabbit Cytokine Array (QAL-CYT-1, RayBiotech, Norcross, GA, USA).

### Statistical analysis

The SPSS 19.0 software (IBM Corp., Armonk, NY, USA) was used for data processing. Measurement data were expressed as mean ± standard deviation. Comparisons between the groups were conducted using one-way analysis of variance (ANOVA) and paired t-test for continuous variables within the groups. *p* < 0.05 was considered to indicate a statistically significant difference.

## Results

### Clinical observation

**Fig. 1:**
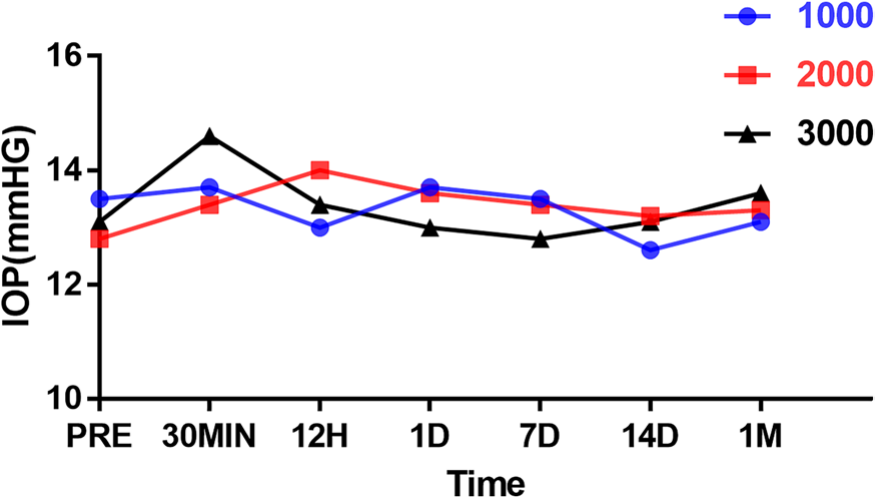
Mean IOP of different study groups recorded at different times (n = 3). IOP, intraocular pressure; PRE, preoperatively; 30MIN, 30 min postoperatively; 12H, 12 h postoperatively; 1D, 1 day postoperatively; 3D, 3 days postoperatively; 7D, 7 days postoperatively

**Fig. 2:**
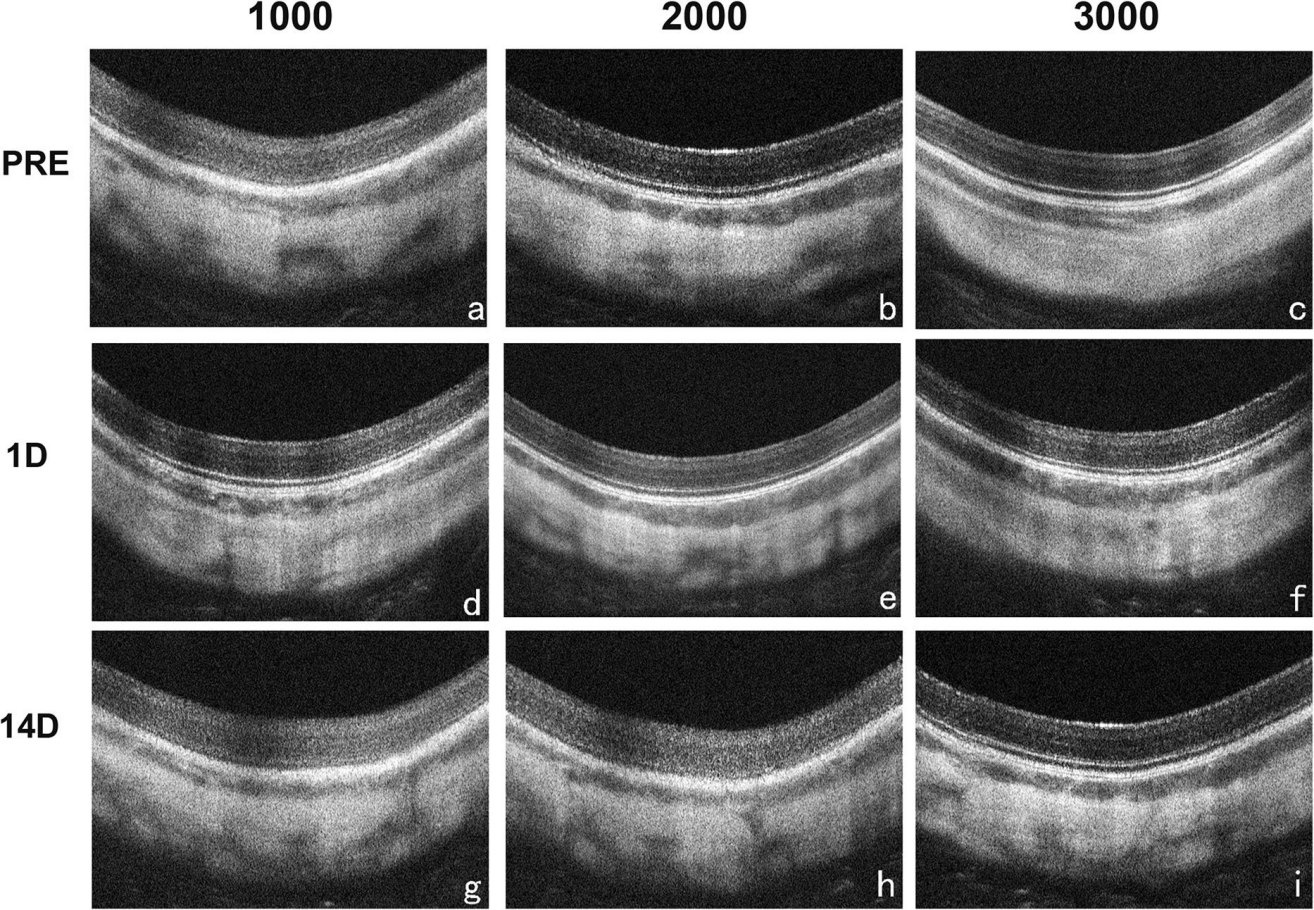
OCT images of different study groups at different times (n = 3). PRE, preoperatively; 1D, 1 day postoperatively; 14 D, 14 days postoperatively

The mean preoperative IOP were comparable among Group 1000 (13.5 ± 1.3 mmHg), Group 2000 (12.8 ± 1.0 mmHg), and Group 3000 (13.1 ± 1.1). Figure 1 shows the IOP changes over time among different groups, and there was no significant difference among the three treated groups at each time point. In Group 3000, a slight IOP elevation (11.5%) was observed at 30 min postoperatively versus IOP preoperatively. However, there was no statistical difference (*p* > 0.05) between IOP at 30 min postoperatively and baseline in Group 3000. As shown in Fig. 2, no changes were found on OCT (Fig. 2). During the study period, there were no signs of a cataract, posterior capsule rupture, vitreous hemorrhage, posterior vitreous detachment, retinal break, or retinal detachment.

### Transmission electron micrographs

**Fig. 3:**
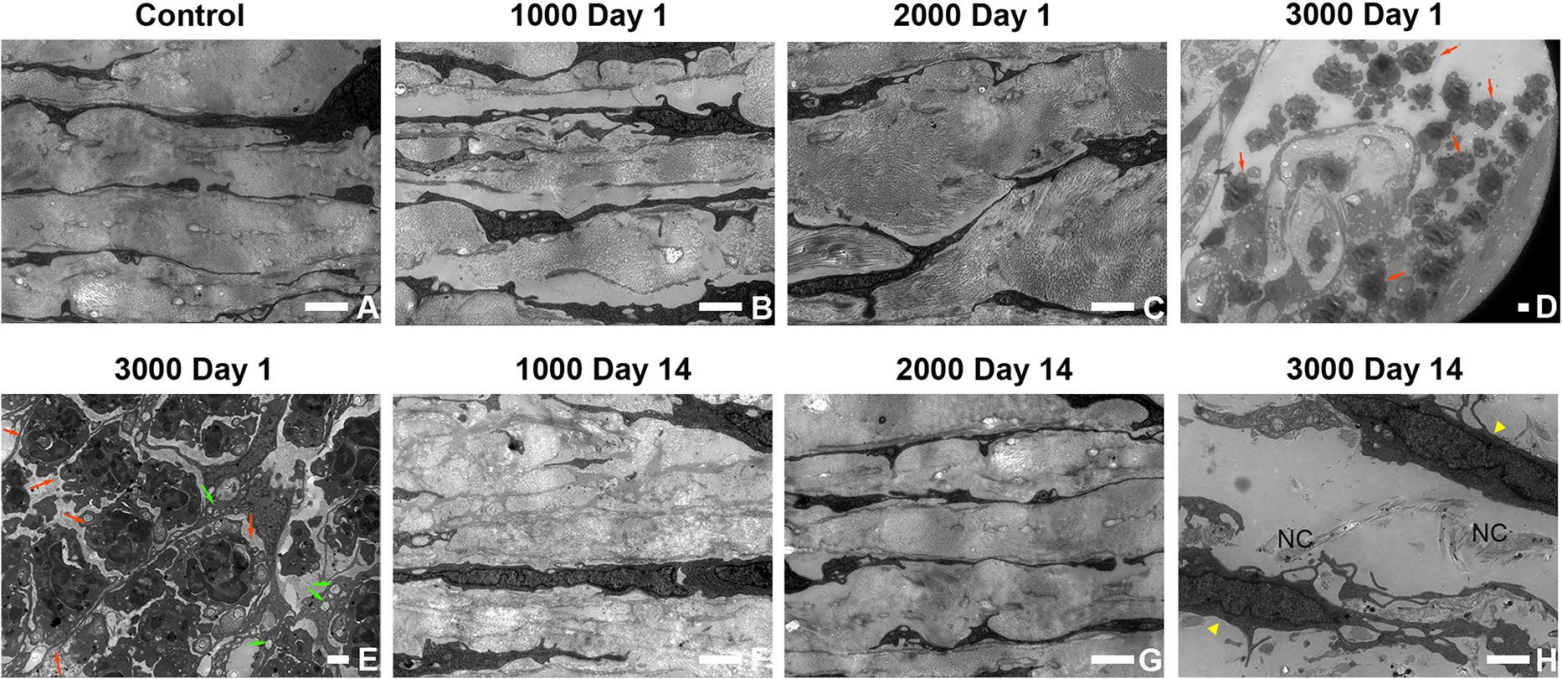
TEM images of trabecular meshwork (TM) and anterior chamber angle of different study groups at different time (n = 3). No abnormalities were observed in control (**a**), Group 1000 (**b**, **f**) and 2000 (**c**, **g**) on day 1 and day 14. Neutrophils and mitochondrial swelling were found in Group 3000 on day 1 (**d**, **e**). Fibroblasts and neocollagen were detected Group 3000 on day 14 (**h**). Day 1, Day 1 postoperatively; Day 14, Day 14 postoperatively; red arrows, neutrophils; green arrows, swelling of mitochondria; yellow arrow heads, fibroblasts; NC, neocollagen. Scale bar = 2 μm

Transmission electron micrographs showed that trabecular meshwork (TM) was normal in the control group (Fig. 3a), Group 1000 (Fig. 3b, f), and Group 2000 (Fig. 3c, g) on day 1 and day 14. Neutrophils were found in the anterior chamber angle of Group 3000 on day 1 (Fig. 3d). Additionally, neutrophils and mitochondrial swelling were also observed in the TM, and the structure of TM was disordered in Group 3000 on day 1 (Fig. 3e) in comparison to the control group and other study groups on day 1. Fibroblasts and neocollagen were detected in Group 3000 on day 14 (Fig. 3h), indicating tissue repair of TM.

### Inflammatory cytokines of aqueous humor quantification

**Fig. 4:**
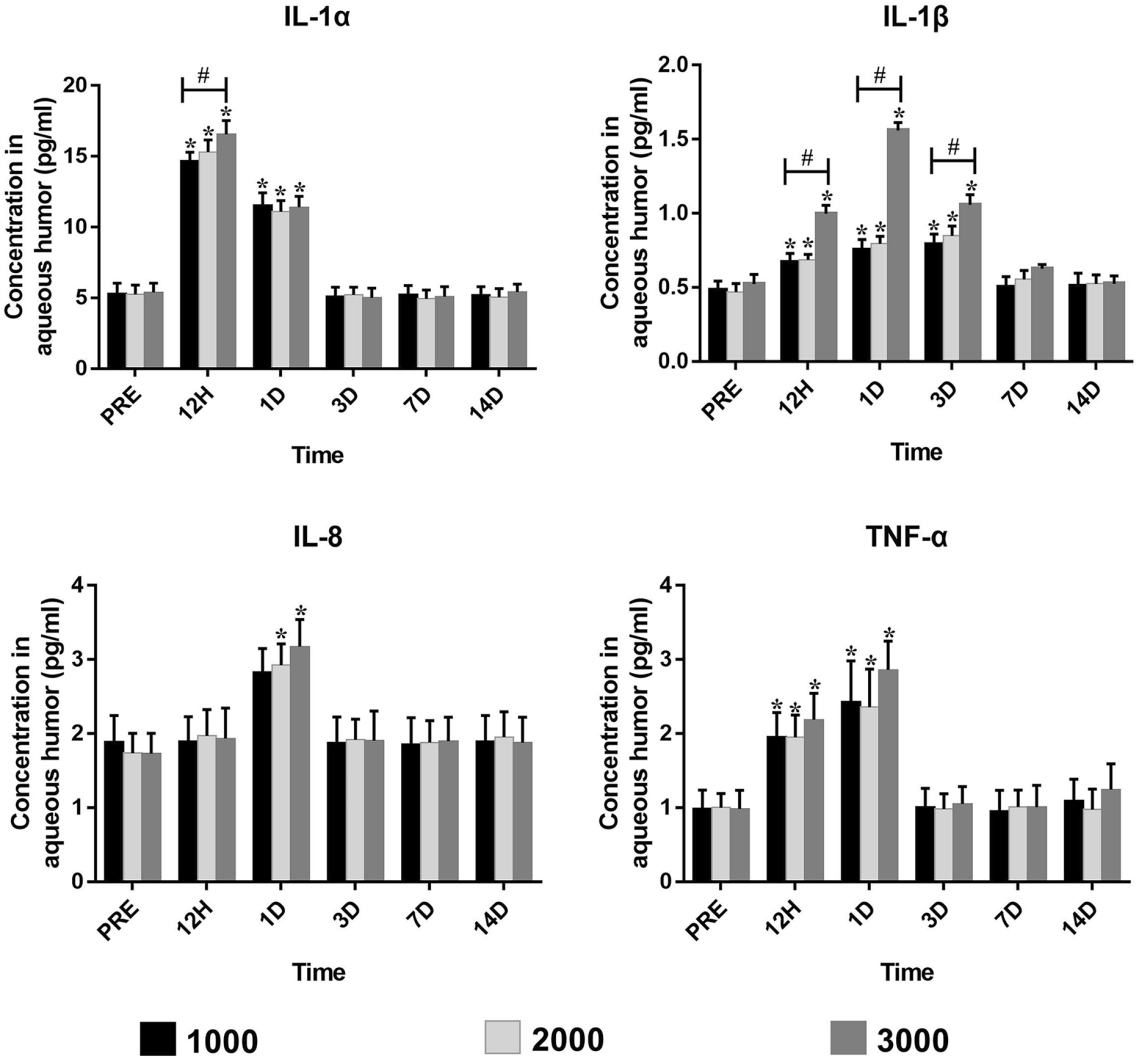
Inflammatory cytokine levels of IL-1α, IL-1β, IL-8, and TNF-α in the aqueous humor before and after YAG laser vitreolysis. *p < 0.05 vs. baseline, # *p* < 0.05 among three study groups at the time point. IL-1α, interleukin-1α; IL-1β, interleukin-1β; IL-8, interleukin-8; TNF-α, tumor necrosis factor-α

Levels of IL-1α from three study groups increased at 12 h, decreased slightly on day 1, then decreased to baseline levels on day 3 (Fig. 4). IL-1α was significantly higher than baseline in Group 1000, 2000, and 3000 at 12 h (*p* < 0.001, *p* < 0.001, and *p* < 0.001, respectively), as well as on day 1 (*p* < 0.001, *p* < 0.001, and *p* < 0.001, respectively). At 12 h, Group 3000 (*p* = 0.004) was considerably higher than the other two groups. No significant difference was found among the three groups at other time points (*p* > 0.05).

Levels of IL-1β from three study groups slowly increased from 12 h to day 3, then decreased to baseline levels on day 7 (Fig. 4). IL-1β was significantly higher than baseline in Group 1000, 2000, and 3000 at 12 h (*p* = 0.001, *p* < 0.001, and *p* < 0.001, respectively), as well as on day 1(*p* < 0.001, *p* < 0.001, and *p* < 0.001, respectively) and on day 3 (*p* < 0.001, *p* < 0.001, and *p* < 0.001, respectively). No significant difference was found among the three groups at other time points (*p* > 0.05).

Levels of IL-8 from three study groups increased on day 1, and decreased to baseline levels on day 3 (Fig. 4). IL-8 was significantly higher than baseline in Group 2000 and 3000 on day 1 (*p* < 0.001 and *p* < 0.001, respectively). No significant difference was found among the three groups at other time points (*p* > 0.05).

Levels of TNF-α from three study groups increased from 12 h, and continued to elevate on day 1, then decreased to baseline levels on day 3 (Fig. 4). TNF-α was significantly higher than baseline in Group 1000, 2000, and 3000 at 12 h (*p* < 0.001, *p* < 0.001, and *p* = 0.002, respectively), as well as on day 1 (*p* = 0.004, *p* < 0.001, and *p* < 0.001, respectively). No significant difference was found among the three groups at other time points (*p* > 0.05).

## Discussion

Noristani et al.^21^, Koo et al.^17^, and Sun et al.^19^ all reported cataract formation following YAG laser vitreolysis, and posterior capsule rupture was found in the three studies. In two studies of Shields et al.^22^, ^23^, retinal break and vitreous hemorrhage happened after YAG laser vitreolysis. In our study, no cataract, vitreous hemorrhage, or retinal break was observed since all posterior capsules and retinas of the three study groups were not injured. We assume that these postoperative complications might be due to the high energy level and the distance being too close between the floater and the posterior capsule or retina. Therefore, it is necessary to set the appropriate safe distance and energy level.

Nowadays, YAG laser vitreolysis has become the conventional method for symptomatic floaters. A total of four clinical trials of YAG laser vitreolysis reported to date, and the number of their patients was 52^12^, 21^14^, 34 24and 51^13^ respectively. The studies above showed no cataracts, elevated intraocular pressure, retinal tears, retinal detachments, or other significant adverse events. However, some case studies described these postoperative complications respectively^15^, ^17^, ^19^, ^21^, ^25^–^27^. The current study assesses the safety of YAG laser vitreolysis for intraocular tissues using Ultra Q Reflex YAG equipment at various energy levels. No iatrogenic injury on retina or lens was detected in each study group. In addition, the IOP of the three groups was within the normal range. YAG laser vitreolysis could induce the elevation of inflammatory cytokines level. Ultimately, the levels of inflammatory cytokines returned to baseline without any therapy before or on day 7 postoperative.

Cowan et al.^15^ reported three eyes of two patients (one is male and the other is female, both in their 50 s) that received YAG laser vitreolysis for the symptomatic floaters and developed PAG. Moreover, all the eyes had a history of YAG capsulotomy for the posterior capsule, and one of the patients received a total of 10 J during 6 procedures. De Vries et al.^25^ reported one male patient (in his 50 s) who developed PAG following YAG laser vitreolysis, and the floater was large in size. In order to eliminate the large floater, high energy might be performed during the treatment. However, in our one-month observation of three study groups after YAG laser vitreolysis (Fig. 1), IOP fluctuated within the normal range in all groups and increased transiently (11.5%) at 30 min postoperatively in Group 3000 with no statistical significance (*p* > 0.05). Meanwhile, our results showed that levels of inflammatory cytokines elevated after treatment, and neutrophils were discovered both in the anterior chamber and the TM of Group 3000 on day 1 from the TEM images (Fig. 3d, e). The thermal effect of YAG laser converted the floaters into gas and micro debris. After the vitreous ablation, levels of inflammatory cytokines elevated, which might stimulate neutrophils. We speculate that the posterior capsule defect facilitated micro debris and inflammatory cytokines moving to the anterior chamber and blocking the trabecular meshwork. Nevertheless, no micro debris was seen from transmission electron micrographs. These results support the notion that high energy levels might lead to TM blockage and induce PAG for patients whose anterior chamber angle function is not on a level with the normal ones.

Little et al.^26^ and Delaney et al.^28^ suggested a safety distance of 2 mm from the posterior capsule and the retina. The Treatment Guidelines for the Ellex Ultra Q Reflex laser recommend that it is better to set the energy at 2.5–4.5 mJ and 500 shots as the upper limit for most treatments per session. The axial length of the vitreous chamber of adult New Zealand white rabbits is about 5.7–6.1 mm^29^. As we concentrated in the middle of the vitreous chamber, the distance from the ablation point to both the posterior capsule and the retina was about 2–3 mm. And the highest level of total energy was 3000 mJ (5 mJ × 600 shots) in our study. We hypothesize that the energy of 5 mJ and 600 shots may also be safe for the lens and the retina when the distance to the posterior capsule and retina is about 2–3 mm. Furthermore, it is suggested that A/B ultrasound scan is necessary as an application to measure the distance from the floater to the posterior capsule and the retina before treatment. Nevertheless, surgeons can't ensure a safe distance during the treatment, owing to the sudden movement of patients.

A case report published by Liu et al. 27 was about a 58 years old female patient diagnosed with acute severe rhegmatogenous retinal detachment after 7 days following YAG laser vitreolysis. A partial posterior vitreous detachment was found before the treatment, and the flashing sensation in her treated eye immediately complained after the treatment. We did not detect retinal or posterior vitreous detachment in any study group. We presume that the retinal tear develops as a result of the mechanical effect of the YAG laser, which releases shock waves to pull the peripheral retina, where the posterior vitreous is attached to the retina. High energy is not advised when partial posterior vitreous detachment occurs.

Acute inflammation that develops after YAG laser vitreolysis is associated with both the mechanical and thermal effects that cause shock waves and tissue evaporation. According to our results, various inflammatory cytokines were released in response to vitreous ablation. The cytokines IL-1α, IL-1β, IL-8, and TNF-α serve as up and down regulators of immunological, inflammatory, and injury repair response^30^. It has been demonstrated that these cytokines can interact synergistically via overlapping biological activities in non-ocular tissues^31^, ^32^. Additionally, these cytokines have similar molecular structure and biological properties in humans and rabbits^33^. According to our results of inflammatory cytokines, levels of IL-1α, IL-1β, and TNF-α increased at 12 h postoperatively, and levels of IL-8 increased on day 1 postoperatively. They all decreased to baseline within 7 days after treatment. For IL-1α and IL-1β, Group 3000 elevated more than the other two study groups at some time points (*p* < 0.05). These results indicate that YAG laser vitreolysis is able to increase the levels of the inflammatory cytokines, and the total energy of 3000 mJ (5 mJ × 600 shots) is not recommended for a single treatment procedure.

In summary, energy plays a critical role in the safety of YAG laser vitreolysis. It is safe to conduct the YAG laser vitreolysis when the total energy is less than 2000 mJ for one treatment procedure, under a safety distance of 2 mm from the ablation point to the posterior capsule and the retina. Longer-term observation and more different levels of energy are still needed.
